# Visual Outcomes in Experimental Rodent Models of Blast-Mediated Traumatic Brain Injury

**DOI:** 10.3389/fnmol.2021.659576

**Published:** 2021-04-15

**Authors:** Lucy P. Evans, Ariel M. Roghair, Noah J. Gilkes, Alexander G. Bassuk

**Affiliations:** ^1^Department of Pediatrics, University of Iowa, Iowa City, IA, United States; ^2^Medical Scientist Training Program, University of Iowa, Iowa City, IA, United States

**Keywords:** blast, retina, traumatic brain injury, visual function, bTBI, rodent

## Abstract

Blast-mediated traumatic brain injuries (bTBI) cause long-lasting physical, cognitive, and psychological disorders, including persistent visual impairment. No known therapies are currently utilized in humans to lessen the lingering and often serious symptoms. With TBI mortality decreasing due to advancements in medical and protective technologies, there is growing interest in understanding the pathology of visual dysfunction after bTBI. However, this is complicated by numerous variables, e.g., injury location, severity, and head and body shielding. This review summarizes the visual outcomes observed by various, current experimental rodent models of bTBI, and identifies data showing that bTBI activates inflammatory and apoptotic signaling leading to visual dysfunction. Pharmacologic treatments blocking inflammation and cell death pathways reported to alleviate visual deficits in post-bTBI animal models are discussed. Notably, techniques for assessing bTBI outcomes across exposure paradigms differed widely, so we urge future studies to compare multiple models of blast injury, to allow data to be directly compared.

## Introduction

Cases of traumatic brain injury (TBI) morbidity are increasing as people are more often surviving blast-mediated TBI (bTBI), an injury especially prevalent among military personnel. Over the past two decades, 417,503 U.S. service members sustained at least one TBI as active military (Defense and Veterans Brain Injury Center, 2020) with nearly 2/3 involving an explosive blast (McKee and Robinson, 2014). Unfortunately, bTBIs are typically classified as mild due to the lack of obvious acute macroscopic injury; consequently, affected service members often return to duty prematurely (McKee and Robinson, 2014; Bryden et al., 2019; Regasa et al., 2019). Many neuropathological processes—microvascular injury, axonal injury, and neuroinflammation—can appear in the days to weeks after blast and have long-term effects on physical, cognitive, and emotional health (Hernandez et al., 2018).

Visual impairments are reported by some 75% of TBI patients, including blurry/double vision, difficulties reading, light sensitivity, and decreased peripheral vision (Armstrong, 2018; Frick and Singman, 2019). These visual impairments can arise due to optic neuropathy, axonal injury, and the loss of retinal ganglion cells (RGCs), which transmit visual stimuli to higher-level processing centers in the brain (Sen, 2017). In animal studies, decreased RGC survival and axonal integrity are strongly implicated with the activation of microglia and macrophages, with unregulated oxidative stress further contributing to RGC loss and optic nerve degeneration (Wang et al., 2013; Gupta et al., 2019).

Technological advancements in protective body armor and headgear have improved survival in combat, producing survivors with an increased number of co-morbidities. Polycarbonate eye protection does reduce the number of penetrating eye injuries, but does not prevent closed-globe damage to the eyes after a blast (Cockerham et al., 2011). Additionally, this protective gear in particular is not always worn, as dust and sweat can accumulate and reduce visibility, leaving the eye susceptible to injury (Cockerham et al., 2009). Furthermore, while many types of TBI produce visual impairments in humans, the variations in injury mechanics and pathophysiology necessitate studying blast-induced visual damage as its own entity. Experimental models of bTBI are critical in studying the mechanisms driving visual pathologies and can be utilized to identify and test novel therapeutic targets to prevent long-term visual dysfunction.

In humans, bTBI can be caused by a wide range of severities applied to multiple organ systems, eliciting various reparative responses from the body. Injury severity can depend on the subject’s orientation to the blast wave, location and duration of impact, distance from the source, and protective equipment. It is further complicated by patient demographics such as sex, age, and coinciding co-morbidities (Cernak, 2017; Bryden et al., 2019). Mirroring the complexity of human injury, current murine experimental models vary in terms of injury location, level of protection from the blast wave, and the device and blast magnitude used to administer a bTBI. Many of these successfully model bTBI pathology, but lack of standardization makes direct comparison of data difficult. Here, we review the devices, exposure paradigms, and assessment criteria currently used in rodent models of bTBI-induced visual impairment.

## Materials and Methods

### Literature Search Process and Inclusion Criteria

**FIGURE 1:**
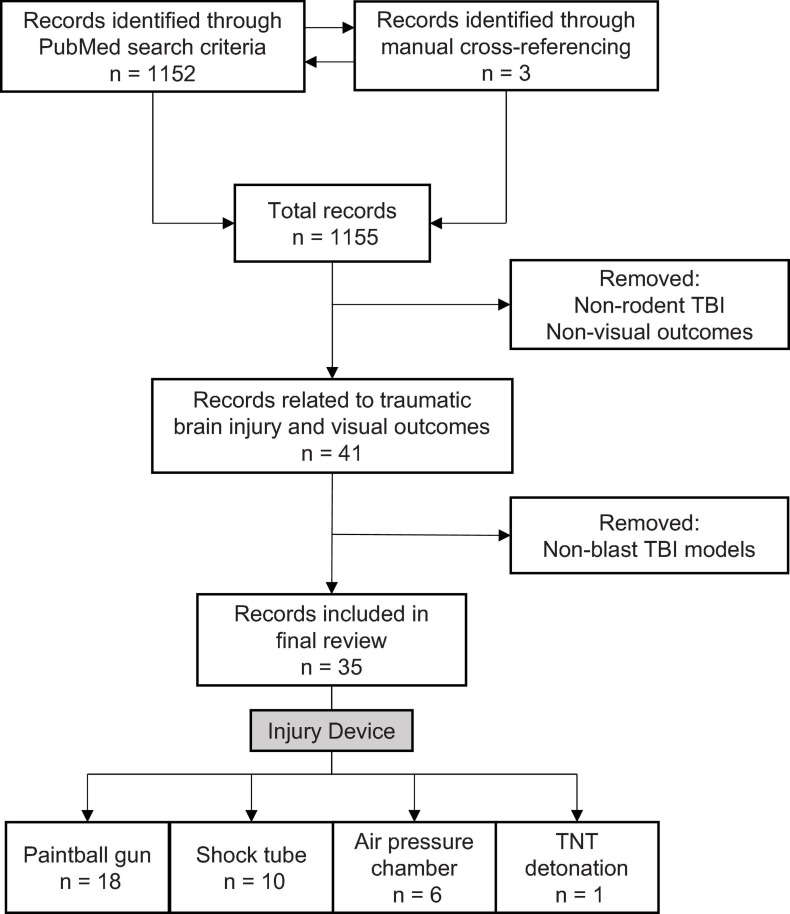
Literature search and selection criteria for rodent models examining blast-mediated TBI and visual outcomes.

To identify relevant literature, we used the default settings on PubMed Legacy edition, using three-part search terms: 1) the subject: mouse/rat/rodent; 2) the injury type: blast/TBI/traumatic brain injury/blast brain injury/brain injury; 3) the visual outcome: eye/vision. Different combinations yielded 28 search terms (e.g., “mouse traumatic brain injury vision”). The process was completed on June 9, 2020, and produced 1,152 results. A manual filtering process was used to exclude non-relevant results, ensuring that the included sources met three necessary inclusion criteria: 1) used a rodent TBI model, 2) evaluated visual outcomes associated with TBI, and 3) employed a blast-TBI model (Figure 1). 35 original literature sources were selected for the review, three from manual cross-referencing.

## Results

### Experimental bTBI Induction

#### TBI Subjects

Although all studies used adult rodent models for bTBI, sex and strain varied. A striking 88.57% (31) of the studies used male subjects only; 8.57% (3) used male and female; and 2.86% (1) used females only. C57BL/6 mice were used in 57.14% (20) of the studies, Long-Evans rats in 14.29% (5), Sprague-Dawley rats in 11.43% (4), Balb/c mice in 5.71% (2), BXD recombinant inbred mice in 2.86% (2), and transgenic mice in 17.14% (6; Table 1).

**TABLE 1:** Transgenic mouse models.

**Transgenic Mouse Strain**	**Clinical Question**
APPswePSENd19e (APP/PS1)	Does AD-like amyloidosis enhance bTBI effects? (Harper et al., 2019a)
EYFP Thy-1 reporter mice (telencephalic neurons)	Histological changes in excitatory neurons after blast? (Reiner et al., 2014; Guley et al., 2016)
Gulonolactone oxidase knockout (Gulo^–/–^)	Effect of enzymatic vitamin C deficiency causing elevated ROS levels? (Bernardo-Colon et al., 2018)
Insulin-like growth factor-binding protein-3 knock down (IGFBP-3 KD)	Protective effect of β-adrenergic receptor activation of IGFBP-3 after blast injury? (Jiang et al., 2014)
Wallerian degeneration slow strain (*Wlds*)	Protective role of nicotinamide mononucleotide adenylyltransferase-1, a catalyst for oxidative phosphorylation, against axonal degeneration after blast? (Yin et al., 2016)

*AD: Alzheimer’s disease; bTBI: blast-mediated traumatic brain injury; EYFP: enhanced yellow fluorescent protein; IGFBP-3: insulin-like growth factor binding protein-3; IGFP-3KD: insulin-like growth factor-binding protein-3 knock down mouse; ROS: reactive oxygen species; *Wlds:* Wallerian degeneration slow mouse.*

#### Blast Injury Devices

**FIGURE 2:**
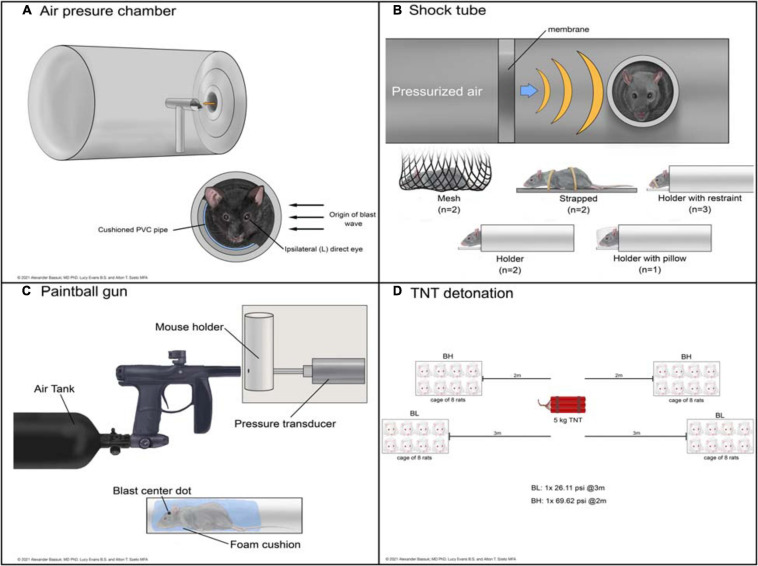
Schematic representations of devices used to deliver bTBI in rodent models. Blast waves were generated by an air pressure chamber **(A)**, through a shock tube **(B)**, from a paintball gun **(C)**, or by TNT detonation **(D)**. (BH): blast high; (BL): blast low.

Devices used to administer bTBI fall into four classes (Figure 2): 51.43% (18) of studies used a modified paintball gun, 28.57% (10) used a compressed air shock tube, 17.14% (6) used an air pressure chamber, and 2.86% (1) used a TNT detonation model (injury parameters found in Table 2). Importantly, there is no commercially available standardized blast equipment; many groups have built or made adjustments to their own injury-induction device, leading to slight variations even within each sub-type of bTBI. However, the majority of groups studying bTBI in this review generally accept that the blast wave created in their model should mimic the Friedlander waveform, modeling the primary blast exposure dynamics that victims are exposed to in the field (Friedlander, 1946; Kuriakose et al., 2016). The Friedlander waveform consists of the shock front, which is an immediate sharp rise in pressure, followed by the blast wind, which is an exponential decay in pressure (Cullis, 2001). All devices induced bTBI with a pressure wave between 3.916 and 80 psi, but target injury locations varied. Control mice underwent an identical process but were not exposed to a blast wave.

**TABLE 2:** Summary of the parameters used in rodent models of bTBI.

**Device**	**Animal**	**Injury location**	**Number of injuries**	**Blast magnitude** (**psi**)
Air pressure chamber	Mouse	Left cranium	1× 1× 1× 1× 3× Varied	3.92 (Yin et al., 2016) 20 (Dutca et al., 2014) 20 (Evans et al., 2020) 20 (Harper et al., 2019a) 20 (Mohan et al., 2013) 5 or 20 (Harper et al., 2019b)
Shock tube	Mouse	Right cranium	3×	43.51 (Mammadova et al., 2017)
	Rat	Face/Front	1× or 5× 1×	**10.15** (Choi et al., 2015) **20** (DeMar et al., 2016)
			1× or 5×	**9.86** (Por et al., 2017)
		Left cranium	1× 1×	11.31, 16.96, *23*.79, or 30.89 (Zhu et al., 2019) 39.02 (Evans et al., 2018)
		Right cranium	1× 1× 1×	**20** (DeMar et al., 2016) 33.36 (Shedd et al., 2018) 80 (Allen et al., 2018)
		Left side	1×	**12.04, 15.08–15.95, or 18.71–25.09** (Petras et al., 1997)
		Right side	1× 1×	**12.04, 15.08–15.95, or 18.71–25.09** (Petras et al., 1997) **17.40** (Wang et al., 2014)
Paintball gun	Mouse	Left cranium	1× 1× 1× 1× 1×	50 (Guley et al., 2019) 50 (Honig et al., 2019) 50 (Jha et al., 2018) 50–60 (Reiner et al., 2014) 0–70 (Guley et al., 2016)
		Left + right eye	1× 1×	26 (Jiang et al., 2013) 26 (Jiang et al., 2014)
		Left eye	1× 1× 1× 1× 1× 1× 3× or 6× 3× or 6× 6× 6×	23, 26 or 30 (Bricker-Anthony et al., 2014a) 23.6, 26.4 or 30.4 (Hines-Beard et al., 2012) 26 (Bricker-Anthony et al., 2014b) 26 (Bricker-Anthony and Rex, 2015) 26 (Bricker-Anthony et al., 2016) 26 (Bricker-Anthony et al., 2017) 15 or 26 (Vest et al., 2019) 15 or 26 (Bernardo-Colon et al., 2018) 15 (Naguib et al., 2020) 15 (Bernardo-Colon et al., 2019)
		Right eye	1×	49 (Struebing et al., 2018)
TNT detonation	Rat	Face	1×	**26.11 or 69.62** (Zou et al., 2013)

*Blast magnitudes in bold indicate studies with whole-body blast.*

The air pressure chamber houses the subject lateral to the blast-wave origin and exposes the left cranium to a long-duration (10–15 ms) blast wave between 3.92 and 20 psi (Figure 2A; Goldstein et al., 2012; Mohan et al., 2013). Anesthetized subjects are in a cushioned PVC pipe with their heads unrestrained to allow for full injury to the left side of the head while preventing injury to the right side. The shock tube is a long chamber that generates a short-duration (2–3 ms) blast overpressure wave, ranging from 9.14 to 80 psi, that travels the length of the tube (Figure 2B; Panzer et al., 2012; Swietek et al., 2019). Impact was delivered to the right or left cranium, or left, right, or front side of the body in anesthetized subjects with various levels of protection.

Shock tube devices administer short (2 to 3 ms) blast overpressures (Panzer et al., 2012; Swietek et al., 2019) while air pressure chambers administer longer (10 to 15 ms) biphasic blast overpressures (Goldstein et al., 2012; Mohan et al., 2013). Whether from expanding gaseous chemicals in shock tubes or pressurized air in pressure chambers, both deliver an initial sharp rise in pressure. This difference in achieving air compression causes the peak in overpressure to be slightly delayed in the air pressure chamber compared to the shock tube. As the wave propagates away from its source, the pressure drops in both devices; in a shock tube this drop occurs exponentially, while in an air pressure chamber the drop is biphasic. The shock wave ends with subsonic particle velocities creating a vacuum with slightly negative pressure, until the wave dissipates (Panzer et al., 2012; Mohan et al., 2013; Swietek et al., 2019).

The modified paintball gun air-tank device emits a brief high pressure air blast that can be calibrated to a specific pressure by adjusting the output from a pressurized air tank, striking the target location on the mouse (Figure 2C; Hines-Beard et al., 2012). It administers bTBI with magnitudes between 15 and 70 psi, with blasts directed at the left eye, right eye, or left cranium. Anesthetized bTBI subjects were placed in a small chamber with a foam cushion to prevent secondary somatic injuries. One study from our literature search utilized 5 kg of 2,4,6-trinitrotoluene (TNT) to generate a blast wave and induce bTBI (Figure 2D; Zou et al., 2013). Exposure conditions were manipulated by varying the distance from detonation. At two meters from the source, mice experienced a blast overpressure of 69.62 psi, whereas at three meters blast overpressure was 26.11 psi (blast high [BH] and blast low [BL], respectively). Anesthetized mice were positioned in metal cages facing the detonation site and secured to the cage loosely with Velcro to prevent movement.

Of note, mice were exposed to whole-body blast in only a handful of studies (bolded studies in Table 2), which can also serve as another variable within bTBI models. Damage to other organ systems can cause alterations in the body’s response to injury and subsequently the systemic environment that the brain and eyes are exposed to after blood-brain-barrier and blood-retina-barrier damage due to blast.

##### Overall summary

Mouse models of bTBI include modified paintball guns, air shock tubes, air pressure chambers, or TNT explosives; while these devices vary, many aim to recreate the Friedlander waveform victims of blast injury experience.

### Pharmacologic Interventions

Pharmacologic intervention was the focus of 34.29% (12) of the studies (see Table 3). Of these, 75% (9) reduced inflammation, 33.33% (4) reduced apoptotic pathways, 25% (3) reduced oxidative stress levels, 8.33% (1) decreased excitotoxicity, and 8.33% (1) reduced tryptophan oxidative degradation. Galantamine, an acetylcholinesterase inhibitor, blocked inflammation, oxidative stress, and excitotoxicity (Naguib et al., 2020). Inflammation was blocked with cannabinoid type-2 receptor inverse agonists, raloxifene (Honig et al., 2019) and SMM-189 (Reiner et al., 2014; Guley et al., 2019) the paracrine factors secreted by adipose stem cell concentrated conditioned media (ASC-CCM) pre-stimulated with inflammatory cytokines (Honig et al., 2019) and interleukin-1 receptor (IL-1RI) antagonist anakinra (Evans et al., 2020). Two approaches were used to deliver erythropoietin (EPO) to treat retinal oxidative stress and neuroinflammation. One method injected exogenous EPO into DBA/2J mice, a short-term exposure; and the second delivered EPO via adeno-associated virus (rAAV), a longer-term EPO treatment. Importantly, the rAAV form of EPO had attenuated erythropoietic activity, so it would not stimulate RBC production and trigger retinal oxidative stress (Bricker-Anthony et al., 2017). Apoptosis was decreased by P7C3-S243, an activator of nicotinamide phosphoribosyltransferase (NAMPT) (Dutca et al., 2014). Both apoptosis and inflammation were reduced by the β-adrenergic receptor agonist, Compound 49b (Jiang et al., 2013, 2014). Inflammation and reactive oxygen species (ROS) were reduced by Vitamin E supplements and a ketogenic diet, while a deficiency in vitamin C that elevates inflammation and ROS was used to study how antioxidants might be therapeutic for bTBI (Bernardo-Colon et al., 2018). Finally, a downregulation of a neurotoxic gene encoding kynurenine 3-monooxygenase (KMO) protected blast mice that were preconditioned (exposed to smaller, five psi blasts prior to a higher-intensity 20 psi blast). A KMO inhibitor (Ro-61-8048) blocked oxidative degradation of tryptophan and formation of neurotoxic intermediates (Harper et al., 2019b).

**TABLE 3:** Summary of the pharmacological interventions and outcomes observed following bTBI.

**Intervention**	**Target Effect**	**Route of Admin**	**Timing of treatment**	**Outcomes**
ASC-CCM	• Anti-inflammatory	IV	4 wk prior to injury	• Prevented loss of VA and CS • Decreased retinal inflammatory cytokines/activation of microglia and astrocytes • Partial protection of RGC complex layer (Jha et al., 2018)
Anakinra (Sobi)	• IL-1 receptor antagonist	IP	Daily for 1 wk prior and 3 wk post-bTBI	• Prevented retinal inflammatory cell activation • Decreased ON degeneration • Rescued PERG deficits • Preserved RGC complex layer (Evans et al., 2020)
Compound 49b	• β-adrenergic receptor agonist	Topical eye drops	Post-bTBI • 3, 24, or 72 h, then 1× for 3 days	• Decreased inflammatory cytokines • Reduced apoptotic proteins (Jiang et al., 2013)
				• Decreased inflammatory cytokines and apoptotic factors • Increased levels of IGFBP-3 (Jiang et al., 2014)
EPO DBA/2J mice (Procrit, Ortho Biotech)	• Inhibit apoptosis	IP	Post-bTBI: • 0, 24, and 48 h • 6, 30, and 54 h • 24, 48, and 72 h	• Decreased axonal degeneration • Decreased retinal cell death and ROS • Decreased GFAP reactivity Only when initiated after injury and assessed at later timepoints; treatment before or soon after injury exacerbated outcomes (Bricker-Anthony et al., 2017)
EPO rAAV.EpoR76E Balb/c mice		IM	1 mo prior injury 24 h post-injury	• Decreased axonal degeneration • Decreased retinal cell death and ROS • No difference in GFAP expression when compared to blast controls Only when initiated after injury; treatment before or soon after injury exacerbated outcomes (Bricker-Anthony et al., 2017)
Galantamine (TCI America)	• AChE inhibitor	Oral	Daily for 30 d post-injury	• Decreased inflammatory cytokines, ROS, axonal degeneration, and VEP/ERG deficits (Naguib et al., 2020)
Ketogenic diet (TD.150843, Teklad)	• Anti-oxidant	Oral	2 w pre-bTBI and throughout the experiment	• Decreased inflammatory cytokines • Reduced caspase-1 • Decreased ON degeneration • Preserved VEP amplitudes (Bernardo-Colon et al., 2018)
P7C3-S243	NAMPT receptor agonist	IP	2× daily post-bTBI until study end	• Rescued PERG deficits (Dutca et al., 2014)
Raloxifene	• Cannabinoid type-2 receptor inverse agonist	IP	2 h post-bTBI and once daily for 14 d following	• Improved VA and CS • Decreased light aversion and normalized pupil constriction • Reduced ipRGC abnormalities and IBA-1 • Decreased ON degeneration (Honig et al., 2019)
Ro-61-8048 (Selleckchem)	• KMO gene inhibitor	Oral	3 d pre-bTBI until study end	• Preserved RGC complex layer • Rescued PERG deficits (Harper et al., 2019b)
SMM-189	• Cannabinoid type-2 receptor inverse agonist	IP	2 h post-injury and daily for 13 d following or until study end	• Improved VA and CS • Decreased inflammatory cytokines • Reduced diffuse axonal injury (Reiner et al., 2014) • Reduced CS deficits • Decreased microglia activation, GFAP, and IBA-1 (Guley et al., 2019)
Vitamin E (D04101102, Research Diets)	• Anti-oxidant	Oral	1 mo prior to bTBI until study end	• Decreased VEP deficits • Decreased ON degeneration • Reduced caspase-1 and inflammatory cytokines (Bernardo-Colon et al., 2018)

*The company and product number are listed in parenthesis, when provided. AChE: acetylcholinesterase; ASC-CCM: adipose mesenchymal stem cell concentrated conditioned media; bTBI: blast-mediated traumatic brain injury; CS: contrast sensitivity; EPO: erythropoietin; ERG: electroretinography; GFAP: glial fibrillary acidic protein; IBA-1: ionized calcium binding adaptor molecule 1; IGFP-3KD: Insulin-like growth factor-binding protein-3 knock down mouse; IM: intramuscular; IP: intraperitoneal; (ip)RGC: (intrinsically photosensitive) retinal ganglion cells; IV: intravenous; KD: ketogenic diet; KMO: kynurenine 3-monooxygenase; NAMPT: nicotinamide phosphoribosyltransferase; ON: optic nerve; PERG: pattern electroretinogram; RGC: retinal ganglion cell; ROS: reactive oxygen species; r.AAV.EpoR76E: EPO recombinant adeno-associated virus; VA: visual acuity; VEP: visual evoked potential.*

#### Overall summary

Most pharmaceutical interventions aimed to reduce retinal inflammation or apoptosis after blast injury.

### Structural Outcomes (Supplementary Table 1)

#### The Eye and the Retina

Blast injury resulted in a wide range of pathologic findings, including corneal edema (Hines-Beard et al., 2012; Bricker-Anthony et al., 2014a, 2016; Bricker-Anthony and Rex, 2015), cataracts (Bricker-Anthony et al., 2014a, 2016), corneal epithelial thinning (Bricker-Anthony et al., 2016), retinal pigment epithelial (RPE) vacuoles (Bricker-Anthony and Rex, 2015; Bricker-Anthony et al., 2016), neovascularization (Bricker-Anthony and Rex, 2015; Shedd et al., 2018), abrasions (Hines-Beard et al., 2012; Zhu et al., 2019), vitreous detachments (Bricker-Anthony et al., 2014a; Bricker-Anthony and Rex, 2015; Evans et al., 2018), and hemorrhage (Evans et al., 2018). Additionally, RGC dendritic rearrangement (Dutca et al., 2014), severity-dependent retinal lesioning and disorganization (Zou et al., 2013), and increased retinal pyknotic nuclei were reported (Bricker-Anthony et al., 2014a, b, 2016; Bricker-Anthony and Rex, 2015). One commonality across several exposure paradigms was progressive RGC complex layer thinning (Dutca et al., 2014; Evans et al., 2018, 2020; Jha et al., 2018; Struebing et al., 2018; Harper et al., 2019a, b; Honig et al., 2019). Retinal thickening was observed in three studies, as bTBI was proposed to stimulate astrocytes and increase expression of vascular endothelial growth factor (VEGF) (Zou et al., 2013; Reiner et al., 2014; Allen et al., 2018; Shedd et al., 2018). Additionally, retinal detachment was reported (Bricker-Anthony et al., 2014a, 2016; Bricker-Anthony and Rex, 2015). Treatment with anti-inflammatory anakinra (Evans et al., 2020), anti-inflammatory ASC-CCM (Jha et al., 2018), and blast preconditioning (Harper et al., 2019b) preserved RGC complex layer thickness; the AD model exacerbated RGC complex layer loss (Harper et al., 2019a).

Intraocular pressure (IOP) was measured in several bTBI rodent models, but the direct effects of blast injury on IOP remain unclear as studies reported increased (Dutca et al., 2014; Bricker-Anthony et al., 2016; Shedd et al., 2018; Bernardo-Colon et al., 2019; Zhu et al., 2019), decreased (Hines-Beard et al., 2012), or unchanged (Hines-Beard et al., 2012; Guley et al., 2016; Zhu et al., 2019) IOP measurements. Some studies detected a change and found IOP returned to baseline between 7 and 60 days post-injury (Bricker-Anthony et al., 2016; Bernardo-Colon et al., 2019). There was no clear association with injury location or type, as IOP measurements varied within each sub-group: increased with paintball gun (Bricker-Anthony et al., 2016; Bernardo-Colon et al., 2019), shock tube (Shedd et al., 2018; Zhu et al., 2019), and air chamber (Dutca et al., 2014) decreased with paintball gun (Hines-Beard et al., 2012) no change with shock tube (Zhu et al., 2019) and paintball gun (Hines-Beard et al., 2012; Guley et al., 2016). Additionally, there was no linear association with increased IOP and increased blast intensity.

#### Optic Nerve

Optic nerve degeneration in blast-injured mice was consistently identified (Petras et al., 1997; Mohan et al., 2013; Bricker-Anthony et al., 2014a, b, 2016, 2017; Bernardo-Colon et al., 2018, 2019; Guley et al., 2019; Honig et al., 2019; Vest et al., 2019; Evans et al., 2020; Naguib et al., 2020). The optic nerve was protected pharmacologically with anakinra (Evans et al., 2020), EPO (Bricker-Anthony et al., 2017), raloxifene (Honig et al., 2019), SMM-189 (Reiner et al., 2014; Guley et al., 2019), and galantamine (Naguib et al., 2020) along with dietary changes such as vitamin E supplements and a ketogenic diet (Bernardo-Colon et al., 2018). Again, the AD model of amyloidosis increased optic nerve degeneration after blast injury (Harper et al., 2019a). SMM-189 (Guley et al., 2019) and raloxifene (Honig et al., 2019) may prevent secondary neuroinflammation in the optic nerve. Similarly, galantamine treatment (Naguib et al., 2020), sufficient levels of vitamin C, vitamin E, and a ketogenic diet (Bernardo-Colon et al., 2018) may protect against optic nerve degeneration by decreasing the presence of ROS and the subsequent upregulation of inflammatory cytokines.

#### Higher Visual Loci and Pathways

Blasted rodents demonstrated edematous forebrain enlargement (Guley et al., 2016), cerebral cortical neuronal loss (Petras et al., 1997; Guley et al., 2016; Yin et al., 2016), microglial activation (Reiner et al., 2014), and axonal transport defects (Bernardo-Colon et al., 2019). One study of repetitive 43.51 psi blasts to the right cranium reported significantly decreased glial fibrillary acidic protein (GFAP) protein expression in the prefrontal cortex after 30 days, while they found no difference in protein levels of ionized calcium binding adapter molecule 1 (IBA-1) or phosphorylated tau. This study found striatal neurotransmitter levels unchanged following repetitive injury, suggesting that neurons in this location were not damaged in their model (Mammadova et al., 2017). Likewise, another group reported no histological changes including tissue destruction or inflammatory cell accumulation 24 days after a single 39.02 psi blast to the left cranium (Evans et al., 2018). Of note, these groups did observe retinal changes described in other portions of this review.

##### Overall summary

The majority of structural changes after blast injury were seen in the eye, retina, and optic nerve, with RGC and optic nerve damage frequently reported.

### Objective Functional Outcomes (Supplementary Table 2)

The functional integrity of photoreceptor and retinal bipolar cells is measured by electroretinogram (ERG), recorded as a- and b- sine waves, respectively (Perlman, 1995). These metrics of retinal health and visual ability offer an *in vivo* diagnostic for both murine and human subjects, however, the results in these studies were inconsistent. When compared to baseline and/or sham controls, the a-wave and b-wave amplitudes in bTBI subjects either increased (Bricker-Anthony et al., 2014b; Allen et al., 2018), decreased (Honig et al., 2019; Naguib et al., 2020), remained unchanged (Mohan et al., 2013), or exhibited both decreased or unchanged values (Bricker-Anthony and Rex, 2015; DeMar et al., 2016; Bricker-Anthony et al., 2017; Zhu et al., 2019). These disparities might be caused by variations in light intensity (Bricker-Anthony et al., 2014a), mouse strain (Bricker-Anthony and Rex, 2015), orientation to the blast wave (DeMar et al., 2016), and/or blast magnitude (Zhu et al., 2019). In terms of pharmacological interventions, raloxifene effectively restored ERG amplitudes (Honig et al., 2019), while galantamine partially prevented waveform reductions (Naguib et al., 2020).

While an ERG reflects the ability of the retina to respond to incoming light, a pattern ERG (PERG) is a functional readout of RGC signaling, providing information about visual transduction from the retina to the brain (Bach et al., 2013). Consistently decreased PERG amplitudes were seen in blast-injured rodents, for up to 16 weeks post-injury (Mohan et al., 2013; Dutca et al., 2014; Yin et al., 2016; Harper et al., 2019a, b; Evans et al., 2020). Interestingly, two studies reported temporary recovery in PERG amplitudes: one at 24 h post-injury (Mohan et al., 2013) and another at 4 weeks with a reoccurrence of impaired signaling again at 16 weeks (Dutca et al., 2014). The *Wlds* genotype (Yin et al., 2016), blast preconditioning (Harper et al., 2019b), and P7C3-S243 treatment (Dutca et al., 2014) preserved PERG amplitudes, hypothetically by promoting survival of the visual circuitry. The *Wlds* genotype protects against axonal degeneration and inflammatory proliferation at the site of injury (Yin et al., 2016), while blast preconditioning is thought to upregulate endogenous survival factors or downregulate harmful ones. In retinas of mice preconditioned with a small blast, RNA sequencing showed KMO was downregulated. Mice receiving daily oral treatments of Ro-61-8048, a KMO inhibitor, had improved PERG signaling (Harper et al., 2019b). Similarly, P7C3-S243 might preserve visual system integrity by activating metabolic cofactors (Dutca et al., 2014). The anti-inflammatory drug, anakinra, is also partially protective of impaired PERG signaling after blast injury via IL-1RI antagonism, preventing the propagation of inflammatory signaling through this pathway (Evans et al., 2020). An AD model developed worse PERG deficits after blast exposure, suggesting AD amplifies the pathologic retinal effects of bTBI (Harper et al., 2019a). Notably, all of the studies that measured PERG administered blast injury to the left cranium using an air pressure chamber with relatively low pressures (psi between 3.92 and 20).

Visually evoked potentials (VEP) via flash stimulation were also assessed. The VEP N1 amplitude, an early response to visual stimuli (Creel, 1995), was normalized by galantamine (Naguib et al., 2020) and vitamin E treatment (Bernardo-Colon et al., 2018), suggesting these pharmacologic agents protect against VEP response deficits. One study examining RGC physiology reported temporary, spontaneous hyperactivity at 1 and 16 weeks after one 20 psi blast to the left cranium (Dutca et al., 2014). RGC hyperactivity is linked to photoreceptor dystrophic disorders and can significantly decrease the quality of vision (Barrett et al., 2015).

#### Overall summary

PERG was a functional outcome commonly investigated after bTBI, with many studies describing impaired PERG and RGC signaling due to blast injury. ERG changes were inconsistent when compared between studies.

### Subjective and Behavioral Visual Outcomes (Supplementary Table 3)

Bilateral contrast sensitivity and visual acuity were consistently damaged by blast exposure, despite differences in blast magnitude and injury location (Bricker-Anthony et al., 2014a, b; Reiner et al., 2014; Bricker-Anthony and Rex, 2015; Guley et al., 2016, 2019; Allen et al., 2018; Jha et al., 2018; Shedd et al., 2018; Struebing et al., 2018; Honig et al., 2019). Deficits were reported as early as 1 day (Shedd et al., 2018) and up to 8 months (Allen et al., 2018) following injury. Some groups found contrast sensitivity and visual acuity improved over time (Hines-Beard et al., 2012; Bricker-Anthony et al., 2014b) while others conversely found it declined, particularly with age (Bricker-Anthony et al., 2014a; Bricker-Anthony and Rex, 2015; Allen et al., 2018). Treatment with raloxifene (Honig et al., 2019) and SMM-189 (Reiner et al., 2014; Guley et al., 2019) promoted full recovery of both contrast sensitivity and visual acuity, while ASC-CCM treatment provided partial recovery of both parameters (Jha et al., 2018).

Pupillary light constriction produced contrasting findings. One day post-20 psi blast to the left cranium, diminished pupillary constriction was seen that resolved after 10 months (Mohan et al., 2013). At 7 months post-injury, another group reported elevated pupillary constriction that was normalized by treatment with raloxifene (Honig et al., 2019). AD mice had an impaired pupillary light response, possibly due to amyloid deposits in the retina (Harper et al., 2019a).

#### Overall summary

Contrast sensitivity and visual acuity were frequently impaired after bTBI, while studies produced conflicting data on changes in pupillary light constriction.

### Subjective and Behavioral General Outcomes (Supplementary Table 4)

Spatial-learning and memory was unaffected by bTBI, as assessed via Morris water maze, at 30 days (Mammadova et al., 2017) and Y maze at 3, 6, and 8 months (Allen et al., 2018). At 7 days post-injury, however, significant behavioral deficits were detectable in Barnes maze performance and could be rescued through a *Wlds* genotype, which prevents axonal degeneration and inflammatory infiltration to the injuries (Yin et al., 2016). That this genotype protects learning and memory implies potential for post-bTBI therapies that preserve axonal integrity and prevent inflammatory infiltration. Depressive behavior and contextual fear (at 6 to 8 weeks post-injury) were identified in blast rodents and were alleviated by SMM-189, which prevents blast-induced loss of Thy-1 fear-suppressing neurons (Reiner et al., 2014). Of note, blast-related vision loss can affect the assessment of cognitive function, as visual and spatial cues guide subjects throughout the task for many readouts.

Blast-injured rodents showed marked decreases in motor coordination and activity at acute time points (≤ 14 days) (Reiner et al., 2014; Guley et al., 2016) but other studies showed no deficits 30 days after injury (Yin et al., 2016; Mammadova et al., 2017; Harper et al., 2019a) suggesting that findings resolved over time. SMM-189 treatment (Reiner et al., 2014) and the *Wlds* genotype (Yin et al., 2016) were protective against motor deficits, while an AD model exacerbated deficits (Harper et al., 2019a). In particular, SMM-189 treatment protected corticospinal tract integrity and cerebellar and motivational circuitry (Reiner et al., 2014), while the *Wlds* genotype protected axonal integrity in the brain and spinal cord (Yin et al., 2016). In the AD model, β-amyloid peptide (Aβ), amyloid precursor protein (APP), and tau protein exacerbated bTBI pathologies (Harper et al., 2019a).

#### Overall summary

Studies have found that blast causes deficits in a wide range of behavioral outcomes, but consistent trends and methodology have not been established.

### Inflammatory Over-Activation Following bTBI

Post-bTBI inflammation was consistently detected in the included studies by identifying activated cellular inflammatory modulators or directly measuring inflammatory molecules (Supplementary Tables 1, 5). In the inflamed retina, activated resident immune cells attract peripheral immune cells to the site of injury, amplifying the inflammatory response. Inflammatory cytokine levels rise and immunomodulatory cells are activated, triggering changes in cellular morphology or protein expression (Simon et al., 2017). Various post-bTBI-retina immunohistochemistry data detected inflammation-associated upregulation of IBA-1 (Bricker-Anthony et al., 2016, 2017; Guley et al., 2016), GFAP (Zou et al., 2013; Choi et al., 2015; Allen et al., 2018; Honig et al., 2019), or both (Bricker-Anthony et al., 2014a; Bricker-Anthony and Rex, 2015; Mammadova et al., 2017; Jha et al., 2018; Guley et al., 2019; Evans et al., 2020), markers of microglial and macroglial activation, respectively, suggesting these inflammatory modulators are responding to stress and propagating inflammatory signals post-injury.

bTBI can activate resident immune cells to release pro-inflammatory cytokines, signaling for prolonged retinal inflammation and exacerbating visual damage (Li et al., 2015; Fehily and Fitzgerald, 2017). Normally, IBA-1 is expressed in quiescent microglia, but following a TBI, activated microglia proliferate, migrate to injured tissue, and exhibit morphological changes (Shapiro et al., 2009). Many studies detected upregulated IBA-1 in the bTBI retina, both as an acute and chronic indicator of ocular trauma and stress (Bricker-Anthony et al., 2014a, b; Bricker-Anthony and Rex, 2015; Guley et al., 2016, 2019; Mammadova et al., 2017; Jha et al., 2018; Honig et al., 2019; Evans et al., 2020). The transition of microglia from the pro-inflammatory M1 state to the reparative M2 state is expressed as the M1/M2 ratio. A prolonged M1 state can damage retinal tissues due to its swelling injury response and downstream release of pro-inflammatory cytokines and free radicals (Loane and Kumar, 2016; Fehily and Fitzgerald, 2017). The M1/M2 ratio decreased after treatment with both raloxifene (Honig et al., 2019) and SMM-189 (Guley et al., 2019), suggesting microglia in blast retinas transitioned toward a reparative phenotype after pharmacologic treatment.

Macroglia (astrocytes and Müller glia) are supportive glial cells within the retina. Astrocytes are located throughout the CNS including in the retina, while Müller glia are uniquely retinal (Sofroniew and Vinters, 2010). Together, their activation in the retina promotes reactive gliosis, a beneficial repair mechanism following injury; over-activation, however, can cause glial scarring, (Pekny et al., 2014) disruption of neural plasticity, and damage to visual circuitry (Sardar Pasha et al., 2017). Although healthy astrocytes strongly express GFAP, gliotic changes and astrocyte hypertrophy can induce pathological levels of GFAP expression. Müller glia significantly increase GFAP expression due to retinal stress; high GFAP expression in Müller glia and their processes is indicative of injury (Eisenfeld et al., 1984). bTBI retinas consistently demonstrated increased GFAP immunoreactivity, suggestive of macroglial activation in this stressed tissue (Zou et al., 2013; Bricker-Anthony et al., 2014a, 2016, 2017; Bricker-Anthony and Rex, 2015; Choi et al., 2015; Mammadova et al., 2017; Jha et al., 2018; Guley et al., 2019; Evans et al., 2020).

Several post-injury drug interventions suppressed activation of microglia and macroglia in the retina. EPO given at least 1 day after injury in DBA/2J mice decreased GFAP expression (Bricker-Anthony et al., 2017). Notably, Balb/c mice treated with rAAV EPO did not show a difference in retinal GFAP between sham and blast, suggesting the timing of EPO therapy is important. ASC-CCM (Jha et al., 2018), anakinra (Evans et al., 2020), and SMM-189 (Guley et al., 2019) downregulated GFAP and IBA-1 expression after blast.

Activated resident microglia and macroglia recruit infiltrating systemic inflammatory cells and upregulate pro-inflammatory cytokines (IL-1α, IL-1β, IL-6, IL-18, IL-33, IFN-γ, TNFα), contributing to retinal pathogenesis, as all of these cell types are activated by and can propagate retinal inflammation (Allan et al., 2005; Holan et al., 2019). Though multiple studies found acute increases in pro-inflammatory cytokines post-blast (Jiang et al., 2013, 2014; Zou et al., 2013; Bernardo-Colon et al., 2018, 2019; Shedd et al., 2018; Struebing et al., 2018; Harper et al., 2019b; Evans et al., 2020) vitamin E was shown to decrease IL-1β (Bernardo-Colon et al., 2018), while ASC-CCM treatment (Jha et al., 2018) and Compound 49b (Jiang et al., 2013, 2014) decreased both IL-1β and TNFα expression. One study also reported galantamine suppressed pathologic elevation in IL-1α and IL-1β (Naguib et al., 2020). Interestingly, retinal expression of IL-1α and IL-1β increased in vitamin C-deficient mice, which had worse post-bTBI outcomes (Bernardo-Colon et al., 2018).

Neutrophil infiltrates were also found in corneal stromal layers following blast injury with concurrent increases in pain and inflammatory signaling mediators such as the transient receptor potential vanilloid 1 (TRPV1) channel, calcitonin gene-related peptide (CGRP), substance P (SP), and endothelin-1 (ET-1). Increases in hematic myeloperoxidase (MPO), a peroxidase enzyme released during the degranulation and activation of neutrophils, were also reported (Por et al., 2017).

Optic nerves of blast-injured mice showed signs of aberrant inflammatory signaling—axonal degeneration (Petras et al., 1997; Mohan et al., 2013; Bricker-Anthony et al., 2014a, b, 2016, 2017; Bernardo-Colon et al., 2018, 2019; Guley et al., 2019; Honig et al., 2019; Vest et al., 2019; Evans et al., 2020; Naguib et al., 2020), microglial activation (Reiner et al., 2014), astrocytic activation/glial scarring (Mohan et al., 2013; Choi et al., 2015; Bernardo-Colon et al., 2019; Vest et al., 2019), and infiltration of CD68-positive cells (indicative of an inflammatory response) (Choi et al., 2015). Infiltration and morphological changes in microglia and astrocytes in the optic nerve appeared to increase acutely, but decrease as early as 1 week post-injury (Reiner et al., 2014; Bernardo-Colon et al., 2019). However, in one study, the astrocyte percent area significantly increased again 30 days after injury, while percent astrocyte parallelism (a measure of astrocyte process orientation and orderliness) remained abnormally low (Bernardo-Colon et al., 2019).

#### Overall summary

Significant evidence in the literature suggests post-blast inflammation contributes to visual dysfunction after bTBI.

### bTBI Upregulates Apoptotic, Necroptotic, and Pyroptotic Mediators (Supplementary Table 6)

bTBI activates programmed cell death pathways, such as apoptosis, necroptosis, or pyroptosis. Caspase activation initiates apoptosis causing DNA cleavage and genome fragmentation (Reed, 2000). Modified DNA is packed into apoptotic bodies that await engulfment by phagocytes resulting in cellular death (Elmore, 2007). The terminal deoxynucleotidyl transferase (TdT) dUTP nick-end labeling (TUNEL) assay detects apoptotic DNA fragments. Caspase-3 functions as an effector caspase, killing the cell by cleaving specific intracellular targets (Pasinelli et al., 2000; Wang et al., 2014). The necroptotic pathway is characterized by plasma membrane rupture that renders the extracellular environment for nearby cells toxic with inflammatory cytokines (Dhuriya and Sharma, 2018; Chen et al., 2019). Necroptosis is associated with the family of receptor interacting protein kinases (RIPs); RIP1 and RIP3 are two critical signaling molecules and markers of necroptosis (Liu et al., 2019). Pyroptosis also features plasma membrane rupture and cytokine release affecting neighboring tissues, but caspase-1 initiates pyroptosis by cleaving the substrate gasdermin D, which creates pores and then ruptures the plasma membrane (Bergsbaken et al., 2009; Man et al., 2017).

Following injury, DNA damage detected via TUNEL assays was suggestive of apoptotic cell death (Jiang et al., 2013; Zou et al., 2013; Bricker-Anthony et al., 2014a, 2016, 2017; Wang et al., 2014; Bricker-Anthony and Rex, 2015). The presence of caspase-3 in the retina (Zou et al., 2013; Bricker-Anthony et al., 2014a; Wang et al., 2014; Choi et al., 2015) and optic nerve (Wang et al., 2014; Choi et al., 2015) after blast was similarly indicative of apoptosis. Additionally, post-bTBI necroptosis was detected via increased retinal RIP1 and RIP3 expression (Bricker-Anthony et al., 2014a, b; Bricker-Anthony and Rex, 2015) while the upregulation of caspase-1 was suggestive of increased pyroptotic activity (Bricker-Anthony et al., 2014a; Bernardo-Colon et al., 2018).

Three studies investigated therapeutic reduction in apoptosis to increase retinal cell survival after blast injury. In the injured retina, Compound 49b stimulated β-adrenergic receptor activation and IGFBP-3 production, which in turn decreased the level of cleaved caspase-3 and decreased TUNEL labeling, reducing retinal apoptosis (Jiang et al., 2013). A ketogenic diet decreased inflammation and ROS levels, leading to a reduction in the level of cleaved retinal caspase-1 following blast (Bernardo-Colon et al., 2018). Finally, an acute increase in EPO in DBA/2J mice exacerbated retinal cell death after bTBI, possibly due to increased oxidative stress from amplified RBC formation and retinal iron levels. However, when analyzed 1 week after injury, this treatment was protective for cell death when compared to controls. EPO treatment with an rAAV with attenuated erythropoietic activity promoted retinal cell survival better if treatment was delayed (Bricker-Anthony et al., 2017).

#### Overall summary

Cell death pathways are frequently implicated in post-blast visual pathophysiology.

## Discussion

We reviewed the devices and exposure paradigms employed in bTBI research and found notable interstudy variations in techniques and assessment outcomes. The variability of bTBI experimental models’ blast magnitude, location of injury, and device biomechanics makes comparing data on visual outcomes difficult; and the picture is further complicated by inconsistencies in outcome measures. Nevertheless, bTBI consistently resulted in increased inflammation, activation of resident inflammatory mediators, impaired PERG signaling, decreased visual acuity and contrast sensitivity, decreased RGC complex thickness, and ON degeneration. This suggests that these characteristics of visual dysfunction after bTBI are reproducible regardless of the technique employed.

Blast injury in humans, as in murine models, is an extremely heterogenous and multifactorial condition that can result in a wide range of consequences. Many of the outcomes measured in animal studies are not practically measured when assessing human injury, i.e., histology or measuring retinal inflammatory modulators at multiple time points. On the other hand, several parameters used to assess human ocular injury cannot be completely recapitulated in murine studies. For example, it is not feasible to measure specific reading issues or subtle changes in color vision in mice. However, the findings in this review of decreased visual acuity, impaired contrast sensitivity, and optic nerve dysfunction have been consistently seen in human blast injury (Cockerham et al., 2009; Scott, 2011; Saunders and Echt, 2012).

While retinal and optic nerve damage was frequently identified after blast injury, many groups did not find overt damage to brain tissue. This lack of consistent changes in the brain after blast could suggest that the retina and optic nerve are more sensitive indicators of mild injury in this model. The increased vulnerability of the eye and optic nerve to bTBI specifically is a unique aspect of this injury model, as other types of TBI can experience greater damage to brain tissue. Additionally, as impaired vision can be a confounding factor for cognitive testing, we recommend that future cognitive testing should be done in concert with tests assessing basic visual performance.

The data implicate inflammatory and apoptotic pathways as playing a causal role in long-term visual dysfunction after bTBI and several targeted pharmacological interventions show promise for manipulating those pathways. Generally, inflammatory blockade protected against deficits in contrast sensitivity, visual acuity, and RGC signaling. Additionally, anti-inflammatory agents preserved RGC complex layer thickness and optic nerve integrity. Interestingly, these pharmacologic interventions targeted different portions of the inflammatory response and, in the studies that reported levels of individual inflammatory cytokines, varied in terms of the actual reduction of inflammatory molecules. This could suggest that multiple inflammatory pathways play a role after bTBI and that combination therapy using multiple agents would confer the most retinal protection after injury.

**FIGURE 3:**
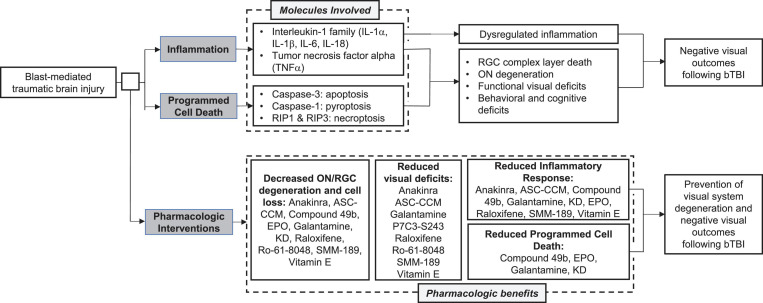
Summary of common molecular pathways, visual outcomes, and pharmacologic interventions following blast mediated traumatic brain injury (bTBI).

It is also clear that programmed cell death contributes to the retinal pathogenesis and subsequent visual disturbances following bTBI. Preventing cellular death is vital for vision preservation and would greatly improve outcomes post-injury. Together, these observations suggest that the overactivation of both inflammatory and apoptotic pathways contribute to visual dysfunction following blast injury (Figure 3). While the field still does not have a gold standard for a rodent blast model, making direct comparisons difficult at times, these common pathways could serve to bridge the gaps caused by variations in experimental techniques and outcome assessments.

Due to the lack of commercially available equipment for blast-induction, standardization of the equipment can be difficult and could contribute to issues with reproducibility across groups using the same type of injury device. However, studies should focus on recapitulating the Friedlander waveform, mimicking the primary blast experienced in the field, enhancing their direct translational potential, and improving cross-model standardization. We recognize the vital need for a study comparing the models directly across a range of blast magnitudes and injury locations to fully understand the commonalities and differences in visual outcomes following varied blast exposure. While this would be a massive undertaking for one group to conduct, the field would benefit greatly from the creation of a large-scale data repository. Individual labs could contribute data generated from their specific parameters, outcomes, and injury type, allowing for comparisons across, as well as within, blast devices, time points, and readouts. This would provide information concerning reproducibility in addition to identifying clear commonalities that could guide research in the search for an effective intervention. We urge that future studies focus on these pathways and their downstream targets to identify specific molecules that could mediate visual protection in patients suffering from bTBI. Experiments pinpointing the anti-inflammatory mediators or survival factors that confer the greatest retinal protection would generate great strides toward translating these treatments to human use.

## Author Contributions

LE, NG, AR, and AB contributed to the conception and design of the study. NG conducted the literature search. LE, NG, and AR wrote sections of the manuscript. All authors contributed to manuscript revision, read, and approved the submitted version.

## Conflict of Interest

The authors declare that the research was conducted in the absence of any commercial or financial relationships that could be construed as a potential conflict of interest.

## Abbreviations

AChE: acetylcholinesterase
AD: Alzheimer’s disease
ASC-CCM: adipose mesenchymal stem cell concentrated conditioned media
bTBI: blast-mediated traumatic brain injury
CE: corneal edema
CGRP: calcitonin gene-related peptide
CNV: corneal neovascularization
CS: contrast sensitivity
dLGN: dorsal lateral geniculate nucleus
ELISA: enzyme-linked immunosorbent assay
EPO: erythropoietin
ERG: electroretinography
ET-1: endothelin-1
EYFP: enhanced yellow fluorescent protein
GFAP: glial fibrillary acidic protein
HPLC: high-performance liquid chromatography
IBA-1: ionized calcium binding adaptor molecule 1
IFN- γ: interferon-gamma
IGFBP-3: insulin-like growth factor binding protein-3
IGFBP-3 KD: insulin-like growth factor-binding protein-3 knock down mouse
IHC: immunohistochemistry
IL-1: Interleukin-1
IM: intramuscular
INL: inner nuclear layer
IOP: intraocular pressure
IP: intraperitoneal
(ip)RGC: (intrinsically photosensitive) retinal ganglion cells
IV: intravenous
KD: ketogenic diet
KMO: kynurenine 3-monooxygenase
LGN: lateral geniculate nucleus
MPO: myeloperoxidase
NAMPT: nicotinamide phosphoribosyltransferase
NLRP: nucleotide-binding oligomerization domain
NO: nitric oxide
NOS: nitrous oxide synthase
OCT: optical coherence tomography
ON: optic nerve
ONL: outer nuclear layer
OT: optic tract
PERG: pattern electroretinogram
qPCR: quantitative polymerase chain reaction
RGC: retinal ganglion cell
RIP: receptor interacting protein kinase
RNFL: retinal nerve fiber layer
ROS: reactive oxygen species
RPE: retinal pigment epithelium
r.AAV.EpoR76E: EPO recombinant adeno-associated virus
SC: superior colliculus
SOD2: superoxide dismutase 2
SP: substance P
TBI: traumatic brain injury
TNF α: tumor necrosis factor alpha
TRPV1: transient receptor potential vanilloid 1
TUNEL: terminal deoxynucleotidyl transferase (TdT) dUTP nick-end labeling
VA: visual acuity
VEP: visual evoked potential
*Wlds*: Wallerian degeneration slow mouse
WT: wild-type
YFP: yellow fluorescent protein.
