# Supplementary material for: Visual Outcomes in Experimental Rodent Models of Blast-Mediated Traumatic Brain Injury
Source: Front Mol Neurosci. 2021 Apr 15;14:659576. doi: 10.3389/fnmol.2021.659576 (PMC8081965; doi:10.3389/fnmol.2021.659576)
Supplement: Supplementary file 1 [file Table_1.pdf]

Supplemental Table 1. Structural Outcomes

**Techniques**	**Timepoint**	**Outcomes**
**A) Eye: Anterior Segment (cornea, anterior chamber, iris, lens)**		
Histology & IHC	0, 3, 7, 14, 28d	Corneal abrasion, persistent CE and central stromal scarring observed following 23.6 psi blast; torn extraocular inferior muscles, corneal abrasion and CE observed following 24.6 psi blast (Hines-Beard et al., 2012)
	0, 3, 7, 14, 28d	No immediate damage observed; from 3d post injury CE, hyphemia, cataracts and CNV were observed (Bricker-Anthony et al., 2014a)
	0, 3, 7, 14, 28, 60d	CE, CNV, cataracts, and corneal epithelial thinning (Bricker-Anthony et al., 2016)
	1, 96h	Neutrophil infiltrates in the corneal stromal layer following 1x and repetitive blast. Increased corneal TRPV1 channel expression following 1x and repetitive blast; increased CGRP, vasoconstrictor ET-1, and SP expression colocalized with TRPV1 expression in the cornea and stromal layer (Por et al., 2017)
	1d, 1, 4, 8wk	Epithelial bullae, stromal inflammation/vascularization, and subepithelial scarring observed (Shedd et al., 2018)
	3, 7, 14, 28d	CE, CNV, calcification, and cataracts observed in **DBA/2J** blast eyes; no anterior injury in C57BL/6 blast eyes (Bricker-Anthony and Rex, 2015)
Intraocular pressure (IOP)	During	Increased IOP during on-going blast with decreased IOP levels in the contralateral eye (Shedd et al., 2018)
	Immediate	No difference in pre- and post-blast IOP (Guley et al., 2016)
	3, 7, 14, 28, 60d	Transiently increased IOP following blast (Bricker-Anthony et al., 2016)
	0, 3, 7, 14, 28d	Decreased IOP following 23.6 psi and 24.6 psi blast compared to baseline; no difference in IOP following 30.4 psi blast (Hines-Beard et al., 2012)
	1, 7, 14, 28d, 8wk	No difference in IOP between baseline and 16.96 or 23.79 psi blast; IOP transiently increased 1wk after 11.31 psi blast; immediately increased following 30.89 psi blast but recovered at 24hr (Zhu et al., 2019)
	1-3d, 7d	Transiently increased IOP following blast that resolved 7d post injury (Bernardo-Colon et al., 2019)
	4wk	**P7C3-S243 did not prevent increased IOP after blast (Dutca et al., 2014)**
**B) Eye: Posterior Segment (vitreous, retina, optic nerve head, choroid)**		
Histology & IHC	97h	Increased GFAP expression following 1x and repetitive blast in the inner plexiform layer & RGC complex layer (Choi et al., 2015)
	1, 12, 24h	Increased 4-Hydroxynonenal, NOS, and beta amyloid immunoreactivity following blast that later resolved to sham levels (Mohan et al., 2013)
	4, 24 hr, 1wk, 1 mo	Blast-induced increase in IBA-1 and GFAP immunoreactivity seen at 4, 24hr, and 1 wk. **Anakinra decreased IBA-1 and GFAP expression at 1 mo post-blast** (Evans et al., 2020)

	24, 72h, 2wk	Increased aquaporin-4 and vascular endothelial growth factor levels; extension of GFAP+ expression in Müller cells; severity-dependent retinal lesioning and disorganization of retinal layers (Zou et al., 2013)
	3d, 2 wk	IBA-1 expression colocalized with degenerating axons following blast that eventually returned to sham levels (Guley et al., 2016)
	3, 7, 14, 28d	Increased pyknotic nuclei in the ONL & INL in both **DBA/2J** and C57BL/6 blast eyes (Bricker-Anthony and Rex, 2015)
	3, 7, 28d	GFAP expression restricted to astrocytes and Müller glia end feet in C57BL/6 blast retinas; GFAP+ Müller glia cell processes observed in **DBA/2J** blast retinas; activated IBA-1+ microglia initially observed in the ONL and INL that was later restricted to the INL (Bricker-Anthony and Rex, 2015)
	3, 7, 28d	Progressively increased INL & ONL pyknotic nuclei; IBA-1+ microglia with an ameoboid-like morphology detected in the ONL of blast eyes (Bricker-Anthony et al., 2014b)
	3, 7, 28d	Increased GFAP+ immunoreactivity in Müller glia cell processes (Bricker-Anthony et al., 2016)
	3, 7, 28d	**Increased acute and decreased chronic GFAP expression in EPO-treated DBA/2J blast Müller glial processes compared to controls; no difference in expression of GFAP+ Müller glial processes between EPO AVV injected Balb/c mice and sham blast controls** (Bricker-Anthony et al., 2017)
	3, 28d	Increased GFAP and IBA-1 immunoreactivity (Bricker-Anthony et al., 2014a)
	3, 7, 30d	**SMM-189 reduced ipsilateral retinal IBA-1 expression levels and extension of GFAP expression** (Guley et al., 2019)
	7, 14, 28d	CD3+, CD4+, CD8+ immune cells observed (Struebing et al., 2018)
	7, 28d	RPE vacuoles detected with increased pyknotic nuclei (Bricker-Anthony et al., 2016)
	14d	More severe right retina histopathologic injury scores reported following face-and right-directed blast compared to sham (DeMar et al., 2016)
	24d	Posterior vitreous detachment, vitreous hemorrhage, inflammatory cell proliferation, photoreceptor degeneration, and RGC loss (Evans et al., 2018)
	4wk	RGC dendritic rearrangement (Dutca et al., 2014)
	4wk	**ASC-CCM prevented RGC complex layer loss following blast** (Jha et al., 2018)
	30d	Decreased ONL thickness in blast ipsilateral retinas compared to sham (Mammadova et al., 2017)
	30d	**ASC-CCM reduced GFAP immunoreactivity and microglial activation following blast** (Jha et al., 2018)
	30d	Increased GFAP, IBA-1, and phosphorylated tau microtubule protein immunoreactivity compared to sham (Mammadova et al., 2017)
	1mo	**Galantamine retained synaptic overlap for photoreceptor ribbon synapses and horizontal cells following blast to preserve visual signaling** (Naguib et al., 2020)
	2mo	Beta amyloid immunoreactivity detected in the RGC complex and plexiform layers in **AD** blast mice; decreased RGC density in **AD** blast mice (Harper et al., 2019a)

	6wk	Decreased number of RGCs after blast (Struebing et al., 2018)
	10wk	Thinning of INL & ONL in ipsilateral retinas following 50-60 psi blast (Guley et al., 2016)
	11wk	**SMM-189 reduced ipsilateral retinal thinning and attenuated contralateral retinal thickening following blast** (Reiner et al., 2014)
	8mo	**Raloxifene restored ipRGC soma size and normalized melanopsin following blast** (Honig et al., 2019)
	8mo	Increased GFAP+ immunoreactivity in Müller glia cell processes (Allen et al., 2018)
	10mo	No difference in retinal structure between sham and blast; minimal reduction in RGC layer cellularity and inflammation following blast compared to sham (Mohan et al., 2013)
OCT	0, 1, 4, 16wk	Progressive RGC complex layer thinning overtime (Dutca et al., 2014)
	1d, 1-8wk	Temporary corneal stromal thickening; bilateral retinal thickening (Shedd et al., 2018)
	1, 7, 14, 28d, 8wk	No retinal detachment, tearing, hemorrhaging, or RNFL/retinal thickening (Zhu et al., 2019)
	3d	Retinal detachment in the midperipheral retina (Bricker-Anthony et al., 2016)
	3, 7, 14, 28d	Acute retinal detachments in blast eyes that mostly resolve overtime (Bricker-Anthony et al., 2014a)
	7d	Disrupted outer segments of the retinal midperiphery and retinal detachments (Bricker-Anthony et al., 2014b)
	14d	Retinal detachments exhibited in C57BL/6 and **DBA/2J** blast eyes (Bricker-Anthony and Rex, 2015)
	28d	No difference between sham and blast central retinas; photoreceptor loss in one blast eye (Hines-Beard et al., 2012)
	4wk	**Anakinra prevented RGC complex layer thinning** (Evans et al., 2020)
	5wk	**The KMO inhibitor Ro-61-8048 restored RGC complex layer thickness to sham levels (Harper et al., 2019b)**
	8 or 16wk	Decreased RGC complex layer thickness following 1x blast and 3x weekly blast; blast preconditioning restored RGC complex layer thickness following blast to sham levels (Harper et al., 2019b)
	2mo	Decreased RGC complex layer thickness in **AD** blast mice (Harper et al., 2019a)
	4mo	Increased retinal thickness in blast eyes (Allen et al., 2018)
	3mo	Decreased RNFL thickness (Mohan et al., 2013)
Retinal permeability	1-20h	**ASC-CCM increased trans-endothelial resistance *in vitro* to preserve retinal barrier function** (Jha et al., 2018)
**C) Optic Nerve**		
	3, 24, 48h	Increased active caspase-3 in blast bilateral ON; undetectable in shams (Wang et al., 2014)

Histology & IHC	97h	Increased ON GFAP expression following 1x and repetitive blast; some CD68+ macrophages detected in the ON (Choi et al., 2015)
	2, 7, 10, 14, 30d	Acute ON axonal degeneration that later decreased with increased astrocyte activation following blast (Bernardo-Colon et al., 2019)
	3, 5, 7d	Acute microglial activation observed in blast ipsilateral ON; **SMM-189 shifted M1 activated microglia to a M2 phenotype; no change in microglia abundance compared to sham** (Reiner et al., 2014)
	3, 5, 7d, 1mo	**SMM-189 decreased axonal bulb abundance and recovered ipsilateral ON axonal loss post-blast** (Guley et al., 2019)
	3, 7, 16d	Swollen axonal bulbs and degeneration in 50-60 psi blast ON; ipsilateral ON degeneration following 0-25 and 40-60 psi blast; decreased ON abundance following blast at later timepoints (Guley et al., 2016)
	7, 14, 30d	Increased acute ON glia following blast trended downward at 7d and 14d, and then increased again at 30d; increased glial parallelism/orderliness along a common axis at 7d (Bernardo-Colon et al., 2019)
	7-14d	Fiber degeneration detected in ipsilateral ON; no contralateral injury (Petras et al., 1997)
	7, 28, 60d	ON axonal degeneration with collapsed and thickened myelin (Bricker-Anthony et al., 2016)
	12d	Blast mice show degeneration of myelin sheath and abnormal structures; ***rescued in WldS mice*** (Yin et al., 2016)
	2, 4wk	**Vitamin E and a ketogenic diet partially prevented axonal degeneration and glial hypertrophy** (Bernardo-Colon et al., 2018)
	2, 4wk	ON axonal degeneration following repeat blast is worse than 1x blast (Vest et al., 2019)
	28d	ON axonal degeneration with chronic collapsed myelin (Bricker-Anthony et al., 2014a)
	28d	Collapsed myelin and axonal degeneration at later timepoints (Bricker-Anthony et al., 2014b)
	28d	No evident axonal injury (Bricker-Anthony and Rex, 2015)
	4wk	**Anakinra decreased ON damage following blast** (Evans et al., 2020)
	1mo	**EpoR76E AAV Balb/c mice injected after blast injury are protected from axonal degeneration when compared to controls** (Bricker-Anthony et al., 2017)
	1mo	**Galantamine prevented ON axon loss following blast** (Naguib et al., 2020)
	2mo	Decreased ON integrity in **AD** blast mice compared to sham (Harper et al., 2019a)
	2, 8, 16mo	**10 mg/kg raloxifene rescued ON axonal loss in the ipsilateral eye** (Honig et al., 2019)
	10mo	Normal structural features in blast ON, but sporadic axonal degeneration and glial scarring (Mohan et al., 2013)
**D) Higher Visual Loci & Pathways**		
Histology & IHC	1, 3d	Increased microglial activation in blast mice in medial & lateral lemniscus, cerebellar peduncles, deep cerebellar white matter & pyramidal tract (Reiner et al., 2014)

	2, 14, 30d	Loss of SC axonal transport(Bernardo-Colon et al., 2019)
	2d-11wk	Edematous forebrain enlargement; neuronal loss in the cerebral cortex; no neuronal injury or GFAP expression in the cerebral cortex (Guley et al., 2016)
	3, 5, 7d	Acute microglial activation observed in blast contralateral OT, SC, & dLGN that later decreases; **<u>SMM-189 shifted M1 activated microglia to a M2 phenotype; no change in microglia abundance compared to sham</u>** (Reiner et al., 2014)
	3, 7, 30d	**<u>SMM-189 reduced IBA-1+ microglial activation in the contralateral OT</u>** (Guley et al., 2019)
	5d, 8mo	**<u>Raloxifene restored the ocular motor area and the extraocular muscles, decreased OT neuronal loss, and partially reversed microglial activation in the OT following blast; 5mg/kg raloxifene decreased IBA-1 immunoreactivity in the OT</u>** (Honig et al., 2019)
	7-14d	Fiber degeneration in the SC, pretectal area, and LGN following blast (Petras et al., 1997)
	12d	***WldS* genotype** rescued axon degeneration in hippocampus, cerebellum, corpus callosum, olfactory bulb, striatum, & thalamus following blast (Yin et al., 2016)
	14d	More severe bilateral OT damage following right-side-directed blast compared to sham; no difference in OT of face-directed blast (DeMar et al., 2016)
	24d	No brain destruction or inflammatory proliferation(Evans et al., 2018)
	1mo	Post-blast histopathologic injury scores were worse compared to sham in the OT, dLGN, SC, occipital cortex (DeMar et al., 2016)
	30d	Decreased GFAP and no change in IBA-1 and phosphorylated tau microtubule protein immunoreactivity in the pre-frontal cortex compared to sham; no changes in striatal neurotransmitters (Mammadova et al., 2017)
	2mo	Increased beta amyloid plaque levels in **AD** blast mice; no difference in neuritic and diffuse plaque load following blast (Harper et al., 2019a)
