# Supplementary material for: Visual Outcomes in Experimental Rodent Models of Blast-Mediated Traumatic Brain Injury
Source: Front Mol Neurosci. 2021 Apr 15;14:659576. doi: 10.3389/fnmol.2021.659576 (PMC8081965; doi:10.3389/fnmol.2021.659576)
Supplement: Supplementary file 2 [file Table_2.pdf]

Supplemental Table 2. Objective Functional Outcomes

Techniques	Timepoint	Outcomes
ERG	7, 28d	No difference in a- and b-wave amplitudes between baseline and C57BL/6 blast; decreased acute a-wave amplitude in **DBA/2J** blast that later recovered with b-wave amplitude remaining decreased over time (Bricker-Anthony and Rex, 2015)
	1, 7, 14d	No difference between sham and face-directed blast; decreased a- and b-wave amplitudes in ipsilateral eye following right-directed blast at 7d (DeMar et al., 2016)
	3, 7, 14, 28d 8wk	No difference in a- and b-waves between sham and 11.31 & 16.96 psi blast; decreased acute amplitudes following 23.79 psi that recovered at 7d; 30.98 psi blast had a bimodal depression at 3d and 8 weeks, with temporary recovery at 7d (Zhu et al., 2019)
	7d	No difference in a- and b-waves(Mohan et al., 2013)
	7, 14, 28d	Decreased a- and b-wave amplitudes following blast for lower light intensities compared to baseline; no difference in a- and b-wave amplitudes for higher light intensities between baseline and blast overtime (Bricker-Anthony et al., 2014a)
	7, 14, 28d	Increased a- and b-wave amplitudes following blast compared to baseline (Bricker-Anthony et al., 2014b)
	1mo	**Galantamine preserved b-wave amplitude following blast** (Naguib et al., 2020)
	6-8wk	**Raloxifene restored peak a- and b-wave amplitudes sham waveforms** (Honig et al., 2019)
	8mo	Increased a- and b-wave amplitudes and delayed implicit timing (Allen et al., 2018)
PERG	1, 12, 24h	Decreased acute contralateral eye amplitudes that later recovered to sham (Mohan et al., 2013)
	4wk	**Anakinra partially rescued amplitudes following blast** (Evans et al., 2020)
	4wk	***WldS* genotype** restored PERG amplitude to sham levels (Yin et al., 2016)
	5wk	**The KMO inhibitor Ro-61-8048 restored PERG signaling to sham levels (Harper et al., 2019b)**
	2mo	Decreased amplitudes in **AD** blast mice compared to sham/WT blast (Harper et al., 2019a)
	16wk	Similarly decreased amplitudes following 1x blast and 3x blast; blast preconditioning restored deficits in amplitudes following blast (Harper et al., 2019b)
	16wk	Decreased amplitude following blast; **P7C3-S243 treatment restored amplitudes to sham levels** (Dutca et al., 2014)
Single RGC physiology multi-electrode array	1, 16wk	Temporary spontaneous hyperactivity in blast RGCs (Dutca et al., 2014)
VEP (flash stimulation)	2, 4wk	**Vitamin E increased N1 amplitude compared to sham and a low-Vitamin C gulonolactone oxidase knockout group** (Bernardo-Colon et al., 2018)
	2, 4wk	Increased N1 amplitude following 1x 26 psi blast at four weeks with decreased N1 amplitude following repetitive blast at two and four weeks compared to sham (Vest et al., 2019)
	1mo	**Galantamine partially increased N1 amplitude following blast** (Naguib et al., 2020)
