# Supplementary material for: Visual Outcomes in Experimental Rodent Models of Blast-Mediated Traumatic Brain Injury
Source: Front Mol Neurosci. 2021 Apr 15;14:659576. doi: 10.3389/fnmol.2021.659576 (PMC8081965; doi:10.3389/fnmol.2021.659576)
Supplement: Supplementary file 3 [file Table_3.pdf]

Supplemental Table 3. Subjective & Behavioral Visual Outcomes

**Techniques**	**Timepoint**	**Outcomes**
Contrast Sensitivity (CS)	1d, 2, 5, 7, 8wk	Decreased CS (Shedd et al., 2018)
	3wk	Decreased CS (Guley et al., 2016)
	4wk	**ASC-CCM treatment prevented deficits in bilateral CS** (Jha et al., 2018)
	4wk	**SMM-189 treatment prevented deficits in bilateral CS** (Reiner et al., 2014)
	1mo	**Raloxifene restored bilateral CS to sham** (Honig et al., 2019)
	2-8mo	Reduced CS in ipsilateral and contralateral blast eyes (Allen et al., 2018)
	6wk	Decreased CS (Struebing et al., 2018)
Light/dark testing	130d	**10 mg/kg raloxifene restored light sensitivity to sham levels** (Honig et al., 2019)
Pupil light reflex	1d, 10mo	Decreased acute pupil constriction that later recovered to sham levels (Mohan et al., 2013)
	2mo	Decreased pupil constriction in **AD** blast mice compared to baseline (Harper et al., 2019a)
	7mo	**5 mg/kg raloxifene partially normalized red light but not blue light constriction; 10 mg/kg raloxifene normalized both to sham** (Honig et al., 2019)
Visual Acuity (VA)	3, 7, 14, 28d	Decreased VA (Bricker-Anthony et al., 2014a)
	3, 7, 14, 28d	Decreased VA following 30.4 psi blast over time (Hines-Beard et al., 2012)
	3, 28d	No difference in VA between baseline and C57BL/6 blast; decreased VA in **DBA/2J** mice following blast (Bricker-Anthony and Rex, 2015)
	7, 14, 28d	Decreased VA (Bricker-Anthony et al., 2014b)
	4wk	**ASC-CCM treatment prevented deficits in bilateral VA** (Jha et al., 2018)
	4wk	**SMM-189 treatment prevented deficits in bilateral VA** (Reiner et al., 2014)
	30d	**SMM-189 treatment restored bilateral VA deficits to sham** (Guley et al., 2019)
	1mo	**Raloxifene restored bilateral VA to sham** (Honig et al., 2019)
	6wk	Decreased VA (Struebing et al., 2018)
