# Supplementary material for: Visual Outcomes in Experimental Rodent Models of Blast-Mediated Traumatic Brain Injury
Source: Front Mol Neurosci. 2021 Apr 15;14:659576. doi: 10.3389/fnmol.2021.659576 (PMC8081965; doi:10.3389/fnmol.2021.659576)
Supplement: Supplementary file 4 [file Table_4.pdf]

Supplemental Table 4. Subjective & Behavioral General Outcomes

Techniques	Timepoint	Outcomes
Balance beam	2mo	Decreased performance in **AD** blast mice compared to WT and **AD** sham (Harper et al., 2019a)
Barnes maze	7d	Deficits in blast mice rescued by ***WldS* genotype** (Yin et al., 2016)
Fear acquisition and extinction test	6-8wk	**SMM-189 restored contextual fear following blast to sham levels** (Reiner et al., 2014)
Foot slip assay	28d	***WldS* genotype** reduced motor coordination deficits following blast (Yin et al., 2016)
Morris water maze	30d	No difference in latency to platform (Mammadova et al., 2017)
Open-field test	1d, 1, 2wk	Decreased performance in a severity dependent manner (Guley et al., 2016)
	1d, 1, 2wk	**SMM-189 prevented deficits in maximum speed and turn radius in open field seen in vehicle treated controls** (Reiner et al., 2014)
	30d	No difference in center times (Mammadova et al., 2017)
Rotarod	1d, 1, 2wk	No significant deficits between sham and 20-40 psi blast; decreased performance that returns to baseline following 50-60 psi blast (Guley et al., 2016)
	1d, 1, 2wk	**SMM-189 decreased rotarod deficits to pre-blast performance** (Reiner et al., 2014)
	30d	No difference in latencies to fall between blast and sham (Mammadova et al., 2017)
Social discrimination test	30d	No difference in time spent in the sniff zone between (Mammadova et al., 2017)
Tail suspension depression test	6-8wk	**SMM-189 rescued immobility following blast to sham levels** (Reiner et al., 2014)
Y maze	6, 8mo	No difference in spatial attention and number of entries over time (Allen et al., 2018)
