# Supplementary material for: Visual Outcomes in Experimental Rodent Models of Blast-Mediated Traumatic Brain Injury
Source: Front Mol Neurosci. 2021 Apr 15;14:659576. doi: 10.3389/fnmol.2021.659576 (PMC8081965; doi:10.3389/fnmol.2021.659576)
Supplement: Supplementary file 5 [file Table_5.pdf]

Supplemental Table 5. Inflammation

**Techniques**	**Timepoint**	**Outcomes**
Cytokine Array	1wk	Inflammatory cytokine increase in vitreous after blast (Shedd et al., 2018)
ELISA	1h	Increased neutrophil peroxidase myeloperoxidase levels in the blood following repetitive blast (Por et al., 2017)
	4, 24, 72h	**Compound 49b decreased TNFα & IL-1β levels when administered 72h after injury** (Jiang et al., 2013)
	4, 24, 72h	**Compound 49b treatment decreased TNFα and IL-1β in IGFBP-3KD mice when administered 24h after injury** (Jiang et al., 2014)
	72h, 2wk	Increased retinal inflammatory cytokines IL-1α, IL-1β, IL-2, IL-4, IL-6, IL-10, IL-12, IL-13, IFN-y, TNFα, GM-CSF and RANTES. Varied based on timepoint and between 26.11 psi and 69.62 psi blast conditions (Zou et al., 2013)
	14, 30d	Increased retinal IL-1α, IL-1β, and IL-2 inflammatory cytokine levels (Bernardo-Colon et al., 2019)
	2, 4wk	Increased retinal IL-1α, IL-1β, and IL-18 following 1x and repetitive blast. **Ketogenic diet decreased retinal IL-1α after 1x and repetitive blast**; insufficient vitamin C caused increased IL-1α, IL-1β, and IL-18 after 1x and repetitive blast (Bernardo-Colon et al., 2018)<br>**Vitamin E decreased retinal** IL-1α and IL-18 levels after 1x and repetitive blast (Bernardo-Colon et al., 2018)
	1mo	**Galantamine decreased IL-1α and IL-1β to sham levels** (Naguib et al., 2020)
qPCR/Microarray	4, 24h	Increased ipsilateral retinal IL-1β, IL-1α, TNFα and IL-6 following blast compared to sham, with all cytokines trending down at 24h except TNFα (Evans et al., 2020)
	1, 3, 5d	**10 mg/kg raloxifene decreased M1 markers and increased M2 markers in the retina and thalamus following blast** (Honig et al., 2019)
	3d, 4wk	**ACS-CCM decreased IL-1β, antigen-presenting cell maker CD86 and CD68+ macrophage expression compared to saline blast** (Jha et al., 2018)
	5d	Differential activation of 13,971 genes. Found three major processes altered after blast: loss of synaptic transmission, impaired cell metabolism, activation of immune system (Struebing et al., 2018)
Western Blot	24, 72h, 2wk	Increased retinal NOS and NO levels stimulate vasodilation and neuroinflammation after blast (Zou et al., 2013)
RNA-Sequencing	5d	Blast preconditioning protects RGC from injury, potentially by decreased expression of KMO (Harper et al., 2019b)
