# Supplementary material for: Visual Outcomes in Experimental Rodent Models of Blast-Mediated Traumatic Brain Injury
Source: Front Mol Neurosci. 2021 Apr 15;14:659576. doi: 10.3389/fnmol.2021.659576 (PMC8081965; doi:10.3389/fnmol.2021.659576)
Supplement: Supplementary file 6 [file Table_6.pdf]

Supplemental Table 6. Programmed Cell Death

**Techniques**	**Timepoint**	**Outcomes**
Caspase-1 (Pyroptosis)	3d	Caspase-1 detected in the ON, INL and RGC complex layer of blast eyes (Bricker-Anthony et al., 2014a)
	4wk	Caspase-1 increased in single and repetitive blast retinas (Bernardo-Colon et al., 2018)
Caspase-3 (Apoptosis)	3, 24, 48h	Caspase-3 detected in bilateral optic nerve, RGC complex layer, and INL in blast eyes (Wang et al., 2014)
	97h	Caspase-3 increased after blast in the RGC complex layer and INL following 1x and repetitive blast; increased in ON for both conditions, but more caspase-3 seen in repetitive blast (Choi et al., 2015)
	3d	Caspase-3 detected in blast ONL (Bricker-Anthony et al., 2014a)
	2w	Increased caspase-3 in neurons in or between outer and inner granular cell bipolar layers, none in RGC and photoreceptor layer (Zou et al., 2013)
RIP-1/3 (Necroptosis)	3d, 28d	Increased RIP-1 and RIP-3 expression in cornea and retina (Bricker-Anthony et al., 2014a)
	3d, 28d	Increased retinal RIP-1 and RIP-3 expression (Bricker-Anthony et al., 2014b)
	28d	Increased retinal RIP-1 and RIP-3 expression (Bricker-Anthony and Rex, 2015)
Oxidative stress	7d	Fluorescent ROS markers CM-H_2DCFDA and dihydroethidium detected with increased cell damage marker nitrotyrosine levels in the inner retina of blast eyes (Bricker-Anthony et al., 2016)
	3, 7d	**EPO increased retinal ferritin in sham and blast mice due to increased erythropoiesis; EpoR76E AAV injection (with attenuated erythropoietic activity) decreased retinal ferritin following blast compared to GFP AVV controls; EPO-treated blast displayed decreased peroxireductases, superoxide dismutases, and antioxidants at later timepoints compared to buffer-treated controls** (Bricker-Anthony et al., 2017)
	1mo	**Galantamine reduced cell damage marker nitrotyrosine immunolabeling in the RGC complex layer following blast** (Naguib et al., 2020)
TUNEL assay	4, 24, 72h	**Compound 49b given within 72h after blast decreased retinal TUNEL labeling** (Jiang et al., 2013)
	48h	Increased TUNEL+ cells in the RGC complex layer and INL of ipsilateral blast retinas (Wang et al., 2014)
	72h, 2wk	Increased acute retinal TUNEL+ labeling following 26.11 psi blast that persisted at 2wk; increased retinal TUNEL+ labeling following 69.62 psi blast that further increased at 2wk (Zou et al., 2013)
	3, 7, 28d	TUNEL+ cells initially seen in the corneal epithelium and stroma in blast mice; retinal TUNEL+ cells observed at all timepoints (Bricker-Anthony et al., 2014a)
	28d	No difference in epithelial cell turnover; increased TUNEL+ labeling in the ONL, and INL/midperiphery in blast retinas compared to sham (Bricker-Anthony et al., 2014b)
